# Deficiency of mature B cells does not alter the atherogenic response to castration in male mice

**DOI:** 10.1038/s41598-022-16846-4

**Published:** 2022-07-28

**Authors:** Anna S. Wilhelmson, Inger Johansson, Linda Fogelstrand, Johan Bourghardt Fagman, Jean-Francois Arnal, Mikael C. I. Karlsson, Åsa Tivesten

**Affiliations:** 1 Wallenberg Laboratory for Cardiovascular and Metabolic Research, Department of Molecular and Clinical Medicine, Institute of Medicine, University of Gothenburg, Gothenburg, Sweden; 2 I2MC, Inserm U1048, CHU de Toulouse and Université de Toulouse, Toulouse, France; 3 Department of Microbiology, Tumor and Cell Biology, Karolinska Institute, Karolinska University Hospital, Stockholm, Sweden; 4 Present Address: The Finsen Laboratory, Rigshospitalet, Faculty of Health Sciences, University of Copenhagen, Copenhagen, Denmark; 5 Present Address: Biotech Research and Innovation Center (BRIC), Faculty of Health Sciences, University of Copenhagen, Copenhagen, Denmark; 6 Present Address: Novo Nordisk Foundation Center for Stem Cell Biology (DanStem), Faculty of Health Sciences, University of Copenhagen, Copenhagen, Denmark; 7 Present Address: Department of Laboratory Medicine, Institute of Biomedicine, Sahlgrenska Academy at University of Gothenburg, Gothenburg, Sweden; 8 Present Address: Department of Clinical Chemistry, Sahlgrenska University Hospital, Gothenburg, Sweden; 9 Present Address: Department of Surgery, Institute of Clinical Sciences, Sahlgrenska Academy, University of Gothenburg, Gothenburg, Sweden; 10 Present Address: Department of Surgery, Sahlgrenska University Hospital, Gothenburg, Sweden

**Keywords:** Lymphocytes, Cardiovascular diseases

## Abstract

Testosterone deficiency in men is associated with increased atherosclerosis burden and increased cardiovascular risk. In male mice, testosterone deficiency induced by castration increases atherosclerosis as well as mature B cell numbers in spleen. As B cells are potentially pro-atherogenic, we hypothesized that there may be a link between these effects. To address whether mature B cell deficiency alter the atherogenic response to castration, we studied B cell-deficient μMT and genotype control male mice on an atherosclerosis-prone *Apoe*^−/−^ background that were castrated or sham-operated pre-pubertally and fed a high-fat diet between 8 and 16 weeks of age to accelerate atherosclerosis development. Genotype did not affect the effects of castration on body weight or weights of fat depots and there were no differences in serum cholesterol levels across the four groups. Atherosclerosis assessed by quantification of lesion area in serial sections of the aortic root was significantly increased by castration and by the μMT mutation, with no significant interaction between genotype and surgery. In conclusion, castration evokes a similar atherogenic response in B cell-deficient μMT and control mice. These data suggest that atherogenesis following castration is unrelated to the effects of androgens on mature B cell numbers.

## Introduction

Testosterone is the most important sex hormone in men, with central roles in reproduction, behavior, and metabolism. Testosterone deficiency in men is associated with increased atherosclerosis burden as well as increased cardiovascular risk^1,2^. In accordance, androgen deprivation therapy, the foundation of treatment of advanced prostate cancer, also has been associated with increased risk of cardiovascular events^3^ and progress in the understanding of the mechanisms underlying castration-induced atherosclerosis will be of great clinical relevance. In line with clinical data, castration *i.e.,* removal of the testes and thereby induction of testosterone deficiency in the male mouse, increases atherosclerosis in animal models^4^.

The immune system is an important player in atherogenesis; many classes of leukocytes mediate and/or modulate an immune reaction against lipid particles in the vascular wall, which is central to the pathogenesis of atherosclerosis^5^. B cells have been shown to have both pro- and anti-atherogenic properties, where both subset and context determine effects on atherogenesis^6^. The role of B cells in atherosclerosis is a highly active research area^6^. Testosterone has important immunological effects, including regulation of B cell homeostasis in both male mice and humans^7,8^. Castration of male mice increases the number of B cell precursors in the bone marrow as well as the number of mature B cells in spleen^7–9^. The castration-induced effects are mirrored by androgen receptor (AR) deficiency; AR-deficient mice show an increase in B lymphopoiesis from the pro-B cell stage in bone marrow through immature transitional B cells to mature B1 and B2 cells in spleen^8,9^.

As castration increases both atherosclerosis and B cell numbers, we hypothesized that there may be a link between these two effects. The aim of the present study was to evaluate a potential role of mature B cells in castration-induced atherogenesis. To address the question, we utilized μMT mice, which lack mature B cells due to a targeted disruption of one of the membrane exons of the immunoglobulin mu chain gene^10^. We crossed μMT mice with atherosclerosis-prone *Apoe*^*−/−*^ mice and studied the atherogenic response to castration in male μMT and control mice.

## Results

To add to our previous detailed investigation of B cell subsets in AR-deficient mice, which are testosterone- and AR-deficient from the embryonal stage and mirror the B cell as well as atherosclerosis phenotype of castrated mice^4,8^, we first asked whether these mice show altered levels of immunoglobulins that are implicated in atherogenesis^11^. Serum total and oxidized low-density lipoprotein (oxLDL)-specific immunoglobulin levels were quantified in male AR-deficient mice (*Ar*^*y/-*^) and controls (*Ar*^*y/*+^) on *Apoe-/-* background that had been fed high-fat diet between 8 and 16 weeks of age. Median [interquartile range] levels of total IgM (*Ar*^*y/*+^ 1.00 [0.83–1.19] and *Ar*^*y/-*^ 0.73 [0.55–1.13] AU, p = 0.25) and IgG (*Ar*^*y/*+^ 1.00 [0.91–1.12] and *Ar*^*y/-*^ 0.83 [0.76–1.00] AU, p = 0.27) as well as oxLDL-specific IgM (*Ar*^*y/*+^ 1.00 [0.82–2.14] and *Ar*^*y/-*^ 0.76 [0.39–1.17] AU, p = 0.16) and oxLDL specific IgG (*Ar*^*y/*+^ 1.00 [0.87–1.49] and *Ar*^*y/-*^ 1.30 [0.83–3.41] AU, p = 0.25) were all unchanged in these mice.

**Figure 1:**
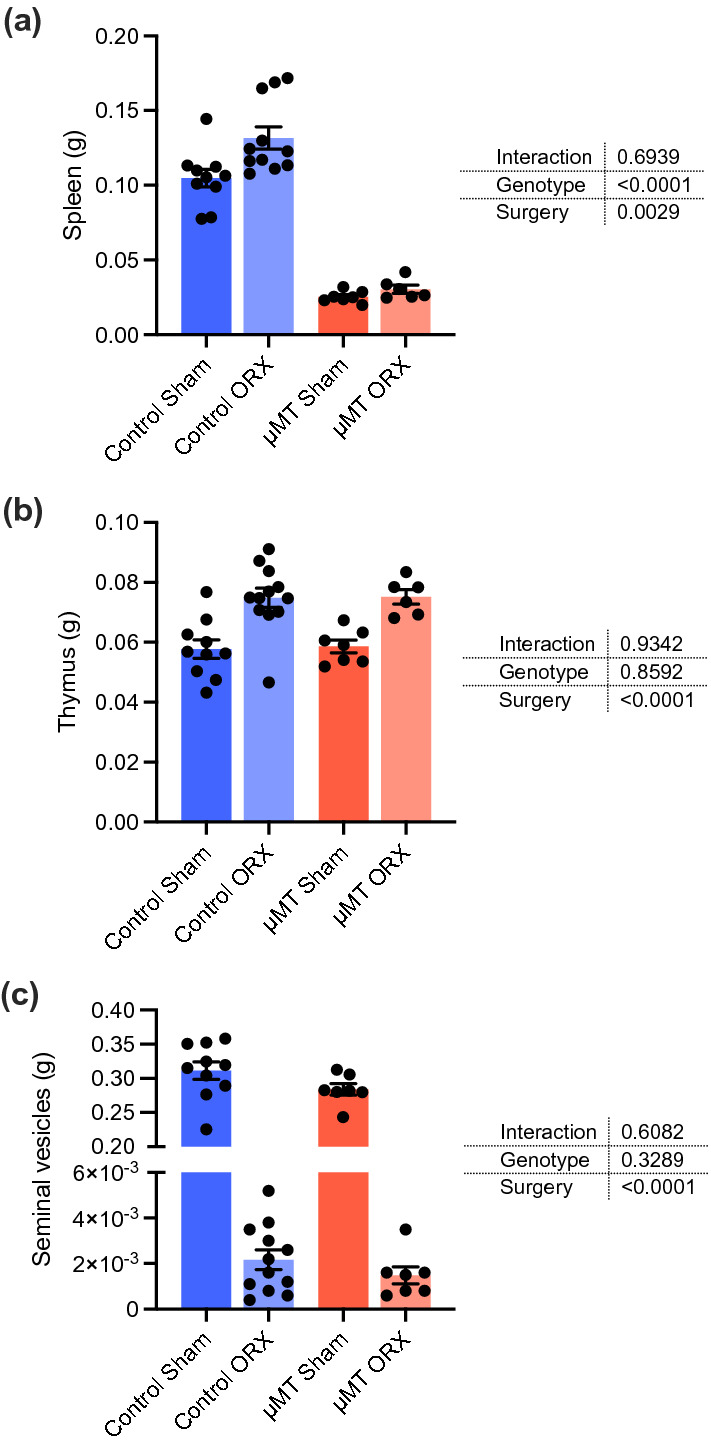
Weights of immunological and androgen-sensitive organs in the µMT/castration model. µMT and control *Apoe*^*-/-*^ mice were castrated (orchiectomized, ORX) or sham-operated (Sham) at 3 weeks of age and fed a high-fat diet between 8 and 16 weeks of age. The weights of the spleen **(a)**, the thymus **(b)**, and the seminal vesicles **(c)** were recorded at the finish at 16 weeks of age (n = 6–12/group). Data were analyzed by 2-way ANOVA. Bars indicate means, error bars indicate SEM, and circles represent individual mice.

To address whether B cell deficiency alter the atherogenic response to castration in male mice, we studied μMT and genotype control mice on an atherosclerosis-prone *Apoe*^*-/-*^ background that were castrated or sham-operated before puberty (at 3 weeks of age) and fed a high-fat diet between 8 and 16 weeks of age to accelerate atherosclerosis development. At 16 weeks of age, spleen weight was prominently affected by the genotype of the mice (− 75.8 ± 1.4% in μMT Sham vs. Control Sham and − 76.8 ± 2.0% in μMT ORX vs. Control ORX) and slightly increased by castration in control mice (+ 25.6 ± 7.1% in Control ORX vs. Control Sham) (Fig. 1a). The well-described effect of castration to increase thymus weight^12^ was similar in μMT (+ 28.4 ± 4.2% in μMT ORX vs. μMT Sham) and genotype control (+ 29.7 ± 5.6% in Control ORX vs. Control Sham) mice, and the μMT mutation itself did not affect thymus weight (Fig. 1b). Weight of the androgen-sensitive seminal vesicles was also similar in μMT and genotype control mice and equally affected by surgery (Fig. 1c), indicating that testosterone production and action was unaffected by the μMT mutation.

**Figure 2:**
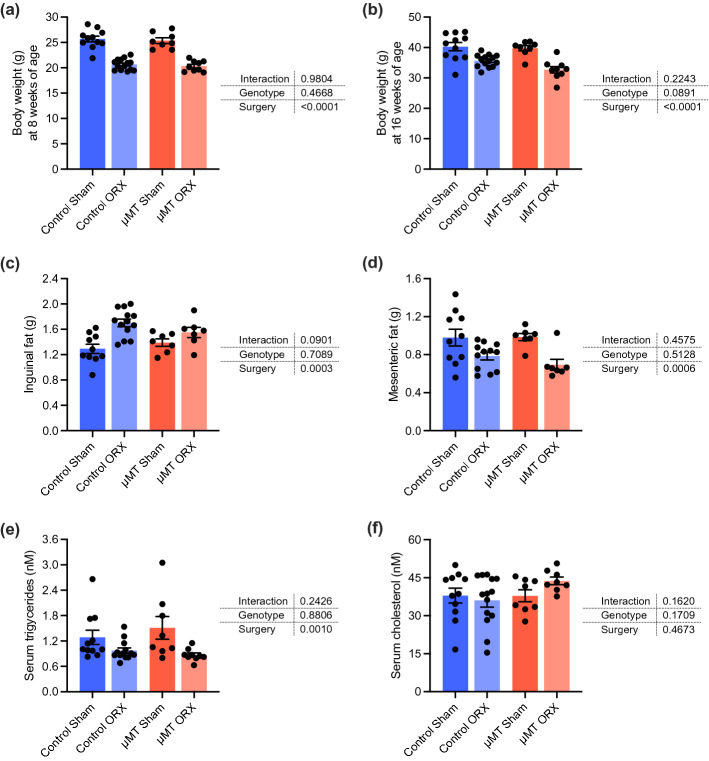
Body weights, weights of fat depots and serum lipids in the µMT/castration model. µMT and control *Apoe*^*-/-*^ mice were castrated (orchiectomized, ORX) or sham-operated (Sham) at 3 weeks of age and fed a high-fat diet between 8 and 16 weeks of age. Body weights were recorded at 8 **(a)** and 16 weeks **(b)** of age (n = 8–14/group). Inguinal **(c)** and mesenteric fat **(d)** depot weights were recorded and serum for triglyceride **(e)** and cholesterol **(f)** measurement were collected at 16 weeks of age (n = 7–14/group). Data were analyzed by 2-way ANOVA. Bars indicate means, error bars indicate SEM, and circles represent individual mice.

Castration reduced body weight, measured both before and after the high-fat diet period (Fig. 2a,b). Further, castration increased weight of inguinal subcutaneous fat and reduced the weight of the mesenteric fat depots as well as the levels of serum triglycerides (Fig. 2c,e). The genotype of the mice did not significantly affect body weight, weights of fat depots or triglyceride levels. Further, genotype did not influence the effect of castration on these variables (Fig. 2a–e). As the main driver of atherosclerosis is cholesterol, we assessed serum cholesterol levels at 16 weeks. However, no significant differences in serum cholesterol levels were detected across the four groups (Fig. 2e).

**Figure 3:**
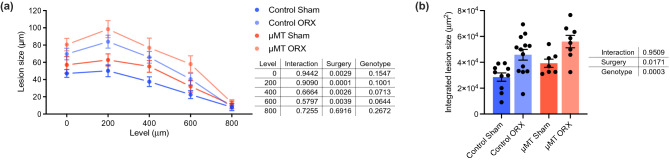
B cell deficiency does not alter the atherogenic response to castration in male mice. Quantification of aortic lesion size in the aortic root of µMT *Apoe*^*-/-*^ that were castrated (orchiectomized, ORX) or sham-operated (Sham) before puberty and fed a high-fat diet between 8 and 16 weeks of age. Lesion size **(a)** displayed per level *i.e.,* distance from the aortic cusps, and integrated estimate of lesion size over levels 0–800 um **(b)** were assessed at 16 weeks of age (n = 7–13/group). Data were analyzed by 2-way ANOVA. Bars indicate means, error bars indicate SEM, and circles represent individual mice.

At the end of the diet period, atherosclerosis was assessed by quantification of atherosclerotic lesion area in serial sections of the aortic root (Fig. 3a). Lesion size integrated over 0–800 μm from the aortic cusps was significantly increased by castration (+ 60.4 ± 14.5% in Control ORX vs. Control Sham and + 42.6 ± 12.2% in μMT ORX vs. μMT Sham) and by the μMT mutation (+ 37.5 ± 11.1% in μMT Sham vs. Control Sham and + 22.3 ± 10.5% in μMT ORX vs. Control ORX) (Fig. 3b), with no significant interaction between genotype and surgery as determined by 2-way ANOVA. Thus, castration-induced atherogenesis was not affected by the μMT mutation.

## Discussion

In this study, we utilized μMT mice that lack mature B cells to explore whether B cell deficiency affects the atherogenic response to castration in male mice. Our main finding is that castration evoked a similar increase in atherosclerosis in μMT and littermate control mice. Further, compared to control mice, male μMT mice showed a small increase in atherosclerosis.

The fact that castration evoked a similar atherogenic response in μMT and control mice suggest that there is no direct link between castration-induced increase in mature B cell numbers and castration-induced atherogenesis. Castration and AR deficiency increases numbers of B cell precursors in bone marrow as well as mature B cells in spleen via two distinct mechanisms^7–9^. In further support of B cell-independent castration-induced atherogenesis, both total and oxLDL-specific immunoglobulins were unaltered in serum of AR-deficient mice that have been shown to mirror the castration-induced B cell and atherosclerosis phenotypes^4,8,9,12^. Mature B cells are deleted in μMT mice as they cannot produce membrane-bound IgM that is needed for their development. In previous studies, neither B cells nor immunoglobulins (total or oxLDL-specific) could be detected in μMT *Apoe*^*-/-*^ mice fed a high-fat diet^13,14^. Spleen weight was reduced by 65% in this μMT setting^13^, as compared to 76% in our study, supporting the validity of our model. Nevertheless, μMT mice have been reported to have small numbers of innate B cells that escape by using another constant chain than mu when challenged^15^. Although these B cells are few, we cannot exclude that they might contribute to regulation of atherosclerosis in the μMT/castration model. Of further note, B cell deficiency may theoretically induce changes in plaque characteristics such as plaque composition and macrophage infiltration, and it is a limitation of our study that these variables have not been addressed. However, while castration increases atherosclerotic lesion area, our previous work has shown that it does not evoke changes in relative plaque collagen or macrophage content in the atherosclerosis setting used in the present study^4^.

Castration also affects T cell homeostasis and we recently showed that castration-induced atherogenesis was blocked by a T cell depletion regimen^12^. Further, male mice with depletion of the AR specifically in epithelial cells showed increased atherosclerosis that was abolished by prepubertal thymectomy, indicating that the thymic epithelial cell is a target cell for the anti-atherogenic actions of testosterone^12^. Notably, here the effect of castration on thymus weight was equal in μMT and genotype control mice. Although T and B cells often act in concert, our data indicates that the castration-induced pro-atherogenic effect on thymic epithelial cells and T cells is predominantly B cell-independent. Further studies are needed to decipher how B cell deficiency affects castration-induced changes in T cell response, which is beyond the scope of this study.

In the present study, male μMT mice showed slightly increased atherosclerosis compared to littermate controls. Previous data on atherosclerosis development in μMT mice are scarce and conflicting. In contrast to our data, one study reported a 65% reduction of atherosclerosis in intact μMT *Apoe*^*-/-*^ mice^16^. In the same study, transfer of wild-type B2 cells to μMT *Apoe*^*-/-*^ mice increased atherosclerosis^16^. Lethally irradiated, *Ldlr*^*-/-*^ mice injected with bone marrow from μMT donors developed increased atherosclerosis^17^, suggesting a protective action of bone marrow-derived B cells. Further, the spleen has been suggested to host a protective B cell-mediated response in atherosclerosis^18,19^. The role of B cells in atherosclerosis is complex and B cells may be both pro- and anti-atherogenic, depending on subset, stage of disease, and effector functions elicited^6^. Of note, depletion of B cells disrupts the architecture of secondary lymphoid organs including development of certain macrophage populations^20^, which may play a role. The atherosclerosis phenotype of μMT mice in different experimental conditions will require further study.

In conclusion, we show here that castration evokes a similar atherogenic response in B cell-deficient μMT and control mice. These data suggest that atherogenesis following castration is unrelated to the effects of androgens on mature B cell numbers.

## Methods

### Animals

μMT mice^10^, homozygous mutant mice that lack mature B cells (*Ighm*^*tm1Cgn*^, Jackson strain #002,288) were crossed with apolipoprotein E knockout (*Apoe*^*-/-*^) mice (B6.129P2-Apoe^tm1Unc^, Taconic model #APOE). μMT^mut/wt^ *Apoe*^+*/-*^ mice were then intercrossed to generate male homozygous mutant mice (μMT^mut/mut^ *Apoe*^*-/-*^) and male littermate genotype control mice (μMT^wt/wt^ *Apoe*^*-/-*^) that were used in this study. Confirmation of gene disruption was screened by polymerase chain reaction genotyping. The strains were backcrossed into a C57BL/6 background for more than 10 generations. Androgen receptor-deficient (*Ar*^*y/*fl^ *Pgk-Cre*^+^ *Apoe*^-/-^) males and controls (*Ar*^*y/*+^ *Pgk-Cre*^+^ *Apoe*^*-/-*^) were generated and genotyped as previously described^4,21^.

### Study protocol

The mice were housed in a temperature- and humidity-controlled room with a 06:00–18:00 h light cycle and consumed diet and tap water ad libitum. The mice consumed a soy-free diet (#2016; Harlan Teklad) after weaning and a high fat diet (#821424, 21% fat from lard, 0.15% cholesterol, Special Diets Services) beginning at 8 weeks of age. At 3 weeks of age, the mice were anesthetized (isoflurane), either sham-operated or bilaterally castrated (orchiectomized). Postoperatively, the mice were given analgesic subcutaneously (buprenorphine) and antibiotics orally (enrofloxacin; added to the drinking water). At 16 weeks of age, the mice were fasted for 3–4 h and euthanized during anesthesia. Blood was drawn from the left ventricle and the circulatory system was perfused with 0.9% saline (pH 7.4) under physiological pressure. Serum was collected from whole blood and frozen for later analysis. Spleen, thymus, seminal vesicles, and fat depots were dissected, and wet weight was recorded. The heart was dissected, and the aortic root was slowly frozen in OCT embedding medium for later processing. All experiments were approved by the Ethics Committee on Animal Care and Use in Gothenburg and followed the Institute for Laboratory Animal Research Guide for the Care and Use of Laboratory Animals as well as the ARRIVE guidelines.

### Serum total and oxLDL immunoglobulins

Levels of total and specific IgM and IgG directed against copper oxLDL were determined in thawed serum by chemiluminescent enzyme-linked immunosorbent assay (ELISA) as previously described^22,23^, and with purified goat anti-mouse IgG (#M30100, Invitrogen) as coating antibody for total IgG. Samples were diluted 1:300 for IgM, 1:50000 for IgG, and 1:200 for oxLDL IgM and IgG. Results were normalized to median levels in the *Ar*^*y/*+^ mice and expressed as arbitrary units (AU).

### Serum triglycerides and cholesterol

Serum total triglyceride and cholesterol levels were determined using Infinity reagents (TR22421 and TR13421, Thermo Fisher Scientific), according to the manufacturer’s instructions.

### Lesion analyses in the aortic root

Serial 10-μm cryosections were cut distally from the aortic root. The sections (0, 200, 400, 600 and 800 µm after the appearance of the aortic cusps) were stained with lipid-reactive Oil Red O and counterstained with hematoxylin. The sections were then evaluated by a blinded observer. We used morphometric analysis (BioPix Software) to determine the area of the atherosclerotic lesions and length of the external elastic lamina (EEL) after manual delineation. As an estimate of atherosclerotic lesion size, atherosclerotic lesion areas were normalized to EEL length and these values were integrated to quantify the total plaque burden.

### Statistical analyses

Statistical evaluations were performed using GraphPad prism software (version 8.4.3). Variables were tested for normal distribution by Shapiro–Wilk normality test. All variables passed normality with or without log transformation and were analyzed by 2-way ANOVA with genotype and surgery as independent variables or unpaired T test. P values of < 0.05 were considered statistically significant. Data represent mean ± SEM or median [IQR].

## Data Availability

The datasets generated during and analyzed during the current study are available from the corresponding author on reasonable request.
